# Relationship Between Attachment Style in Adulthood, Alexithymia, and Dissociation in Alcohol Use Disorder Inpatients. Mediational Model

**DOI:** 10.3389/fpsyg.2018.02039

**Published:** 2018-10-30

**Authors:** Elżbieta Zdankiewicz-Ścigała, Dawid Konrad Ścigała

**Affiliations:** ^1^Faculty of Psychology, SWPS University of Social Sciences and Humanities, Warsaw, Poland; ^2^Institute of Psychology, Faculty of Applied Social Sciences, The Maria Grzegorzewska University, Warsaw, Poland

**Keywords:** attachment style, alexithymia, dissociation, affective self-regulation, addiction

## Abstract

**Background:** Attachment theory is a widely used framework for understanding emotion regulation as well as alexithymia, and dissociation and this perspective has also been applied to understand alcohol use disorders. Apart from these theoretical suggestions, there has been scarce empirical research on this subject-matter. Therefore, the objective of the present study was to investigate potential associations between attachment style in adulthood, alexithymia, and dissociation in alcohol use disorder inpatients.

**Methods:** The Revised Adult Attachment Scale (RAAS), the Toronto Alexithymia Scale-20 (TAS-20), the Michigan Alcoholism Screening Test (MAST), and the Curious Experiences Survey (CES) were administered to a sample of 97 alcohol use disorder inpatients recruited from drug and alcohol treatment centers in Warsaw, and 104 persons in control groups, Poland.

**Results:** A comparative analysis between the group of alcohol addicts and non-addicts showed statistically significant differences related to: alexithymia, dissociation, and attachment styles. The analysis of models related to the impact of attachment styles on the level of alcohol addiction with regard to a mediatory role of alexithymia and dissociation showed that all models are well fitted to data and statistically significant: intimacy *F*(3.197) = 34.41; *p* < 0.001 and explains 34% (*R*^2^ = 0.3438); depend *F*(3.197) = 36.55; *p* < 0.001, and explains 36% (*R*^2^ = 0.3576); anxiety *F*(3.197) = 34.71; *p* < 0.001, and explains 35% (*R*^2^ = 0.3458) of the variability of alcohol addiction scores. Mediation analysis found that alexithymia and dissociation enhance the fear of intimacy and rejection in a romantic relationship.

**Conclusion:** These findings support the broad attachment theory suggesting that attachment is associated with and predicts alexithymia and dissociation in the sample of substance use disorder inpatients. Alexithymia and dissociation, by inhibiting the processes of emotions’ identification and verbalization, cause that language does not serve for the purpose of changing self or others, as the communication is distorted. Absent are common meanings and accurate mutual understanding in a relationship.

## Introduction

The relation between attachment styles and alcohol addiction, or alexithymia itself and alcohol addiction has been documented significantly well (Thorberg et al., 2009). Alexithymia is defined as a difficulty in identifying and communicating feelings, difficulties in differentiating feeling and somatic sensations of emotional arousal, a diminution of fantasy and imagination, and an externally oriented cognitive style (Nemiah et al., 1976; Taylor et al., 1991). Up to 50% of individuals with alcohol use disorders also have alexithymia, a personality construct hypothesized to be related to attachment difficulties (Thorberg et al., 2011). The research on alexithymia has found significant positive associations between alexithymia, and alcohol problems (Thorberg et al., 2009). The attachment theory is a widely used framework for understanding emotions’ regulation (Bowlby, 1973, 1988), as well as alexithymia, and this perspective has also been applied to understand alcohol use disorders. According to attachment theories, early relationships with important attachment figures are crucial for the development of internal operational models for communication, regulation of emotions, and behavior in social situations (Sroufe and Fleeson, 1986; Bowlby, 1988; Cassidy, 1994; Cooper et al., 1998; Laible, 2007; Mikulincer and Shaver, 2007). Attachment styles created in early childhood are strongly connected with those which are presented by people in adulthood in close romantic relationships. Bonds in adulthood are relatively constant and stable, and they influence attitudes, emotions, self-regulation processes, and behavioral strategies in romantic relations (Hazan and Shaver, 1987; Mikulincer et al., 2003; Gillath and Shaver, 2007; Mikulincer and Shaver, 2007). Data obtained from various research indicate a relation between problems pertaining to understanding and regulating emotions in persons brought up in insecure attachment and alexithymia (Hexel, 2003; De Rick and Vanheule, 2006, 2007; Bekker et al., 2007), and between a secure attachment and a higher level of competence, empathy, capability to establish close relationships and emotional awareness and self-awareness (Laible, 2007). Insecure attachment is associated with alexithymia and both harmful drinking (Finzi-Dottan et al., 2003) and substance use disorders (McNally et al., 2003). In addition, alcohol abuse has been hypothesized to be a consequence of alexithymia (Taylor et al., 1997). Another construct relevant to alcohol-dependence is craving characterized by a strong desire for alcohol. It is associated with impaired control over drinking, and includes obsessive thoughts and compulsive drinking behaviors (Addolorato et al., 2005), and plays an important role in the pathogenesis of alcohol-dependence and relapse (Addolorato et al., 2005). Pocuca et al. (2018) carried out the study aimed to determine whether personality profiles moderated the relationship between perceived peer drinking and early adolescent drinking. Baseline data were utilized in the analyses, from 3287 adolescents born in Australia. They found the personality profiles of impulsivity, sensation seeking, and hopelessness to be positively related to early adolescent drinking, whereas anxiety sensitivity had a negative association. These results indicate that the perception of peer drinking is more strongly associated with early adolescent drinking, when adolescents are also high on sensation seeking. Bozkurt et al. (2013) was to evaluate the aggression and impulsivity in two different groups of men with alcohol or heroin dependency. Participants were consecutively admitted male alcohol (*n* = 94) or heroin (*n* = 78) dependent inpatients and healthy controls (*n* = 63). Aggression and impulsivity scores were higher among both the alcohol and the heroin dependent groups than the healthy controls. Verbal aggression was the only subscale, which did not show significant differences between groups. Different dimensions of aggression and impulsivity discriminated these groups from healthy controls. In the next study, Bozkurt et al. (2014) carried out a study, which was to evaluate the relationship of personality dimensions with impulsivity among men with alcohol dependence. Participants were consecutively admitted male alcohol-dependent inpatients (*n* = 94) and healthy controls (*n* = 63). The study was conducted at the end of the detoxification processes of alcohol-dependent inpatients. The severity of impulsivity and dimensions of impulsivity were higher in alcohol-dependent inpatients than in healthy controls. Impulsivity was negatively correlated with reward dependence, persistence, self-directedness, and cooperativeness, but positively correlated with novelty seeking, harm avoidance, depression, and anxiety. In this study, depression, novelty seeking, harm avoidance, and low reward dependence predicted impulsivity. Also, harm avoidance, reward dependence, and self-directedness predicted attentional impulsiveness, depression, and novelty seeking predicted motor impulsiveness, and low cooperativeness and persistence predicted non-planning impulsivity. Thus, an important finding of this study is that personality dimensions that predict impulsivity seem to differ according to the dimension of impulsivity that is evaluated. Each dimension is influenced by complex interactions among genetic and environmental variables because individual personalities develop as complex adaptive systems. In response to stress or environmental cues, an individual with alcohol dependency could use the alcohol in a rapid unplanned action regardless of the consequences. Martinotti et al. (2008) carried out a study which was to investigate the relationship between anhedonia, craving, and temperament and character dimensions in a population of patients with alcohol and opiate dependence. They assumed that some personality traits may be associated with anhedonia and craving symptoms after detoxification, which in turn, would increase the risk of relapse. Important in this study is the fact that the clinical assessment of subjects was performed after approximately 10 months of abstinence. In addition, the minimum time since last detoxification was 90 days. Therefore, ratings were not confounded by intoxication and acute withdrawal. The most important result of the study is the positive correlation between anhedonia and novelty seeking in addiction people. They also found the higher score of novelty seeking in the anhedonic subjects. Martinotti et al. (2008) concludes that hypothesis that difficulty in experiencing pleasure in psychiatric disorders can lead to the use of psychoactive substances in an attempt to decrease anhedonia may be extended to subjects without psychiatric disorders, who may try substances to counterbalance a tonic state of anhedonia. In another study by Martinotti et al. (2009), the purpose was to evaluate the level of empathy in a sample of alcohol-dependent patients in comparison to a control sample. All the subjects successfully detoxified have been evaluated with the empathy quotient (EQ) and then compared with 107 control subjects. The level of empathy was significantly lower in the group of alcohol-dependent subjects than in the control sample. Interesting in this research is the fact that there were no significant differences between the subgroups of alcoholics with and without an Axis II disorder. An impairment in the ability to understand the mental and affective state of others could be at the root of the high levels of impulsivity, aggression, and antisocial behavior, which represent key characteristics of some typologies of alcoholics. The role of alexithymia is another factor to be taken into account. Because the awareness of one’s emotional states is a prerequisite to recognizing such states in others, alexithymia, or the difficulty in identifying and expressing one’s own emotional states, presumably involves impairment in the ability to feel empathy. Thorberg et al. (2011) carried out the study aimed at the relationship between alexithymia, craving, anxious attachment, and alcohol-dependence severity. They found that alexithymia and difficulties in identifying and describing feelings related to preoccupation, obsessions, and compulsive behaviors regarding drinking in those with alcohol-dependence, and alcohol-dependence severity. Alexithymia and insecure attachment were associated with more intrusive and interfering cognitions, ideas, and impulses about alcohol, including an impaired ability to control these thoughts and impulses.

As it was proven, attachment styles may foster forming an intrapsychic ground for the development of emotional skills (Maruszewski and Ścigała, 1998) or to block them (Picardi et al., 2005; Meins et al., 2008). The obstruction resulting from the application of invasive defense techniques against experiencing strong negative emotions leads to the development of alexithymia, and thereby to the deficits of self-regulation (Wearden et al., 2005). This is indicated in the study carried out by Fossati et al. (2009) and concerning the relation between attachment styles, alexithymia, and an inclination to impulsive aggression. The strongest proved to be the mediatory effect of the dimension of alexithymia, i.e., difficulties in identifying emotions, on an inclination to impulsive behaviors. The authors suggest that difficulties in creating mental representations of affective states in persons with a high level of alexithymia may be related to experiencing intensive overwhelming emotions, and not merely to the absence of an ability to identify them cognitively and perceive consciously. Evren et al. (2015) evaluated the relationship between alexithymia and aggression among men with substance dependence. In addiction, they control the effect of variables such as depression and anxiety on this relationship. They found that the alexithymic group had higher scores of aggression, depression, as well as trait and state anxieties, and the difficulty in identifying feelings factor of alexithymia was related to aggression, although chronic anxiety contributed to this relationship particularly in the anger and hostility dimensions of aggression. However, the fact that it is so difficult for persons with a high level of alexithymia to feel emotions, and further to verbalize them, increases their intensity undoubtedly, and thereby the overload with a difficulty to overcome unspecified affect (Lyvers et al., 2017). Several studies have reported that alexithymia is associated with deficits in the ability to recognize and label facial expressions (Grynberg et al., 2012) of both positive and negative emotions, which may be linked to problems with empathy (Grynberg et al., 2010; Valdespino et al., 2017; Lyvers et al., 2018); and theory of mind (Kopera et al., 2014), and problems between couples and co-dependent dynamics (Fischer and Wiersma, 2012).

Recently, alexithymia has been examined as a possible predictor of dissociative tendencies (Zlotnick et al., 1996). Dissociation may interfere with the connections between affects, cognitions, and voluntary behavior control by influencing the development of alexithymia, and resulting in the dissociation of the physiological, cognitive, and affective components of emotions. Both dissociation and alexithymia have been considered impairments of emotive perception that help trauma survivors manage overwhelming or difficult affective states. The study carried out by Craparo et al. (2014), Zdankiewicz-Ścigała (2017), and Zdankiewicz-Ścigała and Ścigała (2018) aimed to explore the incidence of early trauma, alexithymia, and dissociation and to better understand the interaction between alexithymia, dissociation, and trauma in a sample of individuals with alcohol abuse. This population was selected because several studies have found that alcohol-dependent individuals often present a history of early trauma (mainly childhood abuse and neglect) in conjunction with alexithymic traits and dissociative tendencies.

No research has been carried out which would take into account the simultaneous impact of attachment styles, alexithymia, and dissociation, on the inclination to alcohol abuse in adulthood. The purpose of the presented study is to verify a direct impact of attachment styles on the level of alcohol addiction with regard to indirect effects of alexithymia and dissociation.

## Materials and Methods

This study was carried out in accordance with the recommendations of the SWPS University of Social Sciences and Humanities Ethics Committee with written informed consent from all participants. All procedures performed in studies involving human participants were in accordance with the ethical standards of the institutional and/or national research committee and with the 1964 Helsinki declaration and its further amendments or comparable ethical standards. An ethics approval for this research was not required as per the SWPS University of Social Sciences and Humanities Ethics Committee’s guidelines and national regulations. Standardized questionnaires, which are used in psychological worldwide research, were exclusively used in the research procedure. This type of research is based on guidelines and procedures in accordance with applicable law and ethics, but do not require individual consent. Consent to the study was approved by the appropriate authorities of the therapeutic departments and the patients themselves. It was carried out in addiction therapy departments by psychologists working permanently with patients. Before starting to fill in the questionnaires, they were asked to sign an informed consent form which specified all their tasks and rights. The study was carried out among 97 patients of three addiction treatment centers from the 8-week abstinence-based inpatient treatment program combined intensive group and individual therapy as well as elements of 12-step facilitation and relapse prevention, and 104 subjects who have not been treated for alcohol addiction. The study was conducted at the end of the detoxification processes of alcohol-dependent inpatients. The total number of study participants amounted to 201 persons, including 67 women (33.3% of participants) and 134 men (66.7% of participants). The participants aged from 18 to 68 (*M* = 32.81; *SD* = 12.12). Based on a MAST questionnaire for diagnosing alcohol addiction, participants were divided into a control group (a score below 4 points), a group of likely addicted individuals (a score of 4 points), and a group addicted individuals (a score over 5 points). The control group comprised 100 individuals (49.8% of subjects), 40 women and 60 men, at the age of 18–50 (*M* = 25.50; *SD* = 7.40). The group of individuals likely addicted to alcohol comprised four persons (2.0% of subjects), exclusively men at the age of 21–27 (*M* = 23.25; *SD* = 2.63). The alcohol addicts group comprised 97 persons (48.3% of subjects), 27 women and 70 men at the age of 20 to 68 (*M* = 40.73; *SD* = 11.28). All subjects, who were patients of addiction treatment centers, entered the group of alcohol addicts.

### The Revised Adult Attachment Scale (RAAS)

The Revised Adult Attachment Scale (RAAS; Collins and Read, 1990; Collins, 1996) is an *18-*item measure of adult attachment style. It consists of three subscales: Close, Depend, and Anxiety. The Close subscale measures the level of comfort the individual feels with closeness and intimacy. The Depend subscale assesses if the individual feels they can depend on others to be available when needed. The Anxiety subscale measures the level of anxiety the person feels about being rejected or unloved. High scores on Close and Depend, and low scores on the Anxiety dimension, indicate a secure attachment style (Collins and Read, 1990; Collins, 1996). Each item is scored on a five-point Likert scale with some items being reverse scored. The RAAS has demonstrated adequate validity and reliability (Collins and Read, 1990). In the present investigation, the Cronbach alphas were 0.86 for Anxiety, 0.63 for Depend, and 0.56 for Close, respectively. It should be pointed out that the RAAS does not assess attachment styles, but continuous attachment dimensions hypothesized to underlie adult attachment (Bartholomew and Horowitz, 1991; Collins, 1996).

### The Toronto Alexithymia Scale-20 (TAS-20)

The Toronto Alexithymia Scale-20 (TAS-20; Parker et al., 1993) was applied to investigate the level of alexithymia. Apart from the general level of alexithymia, the questionnaire allows to calculate separate scales for the following dimensions: “difficulty describing feelings”; “difficulty identifying feelings”; “externally oriented style of thinking.” The questionnaire comprises 20 test items. Each item has a five-degree Likert scale (1 – strongly disagree; 2 – partially disagree; 3 – undecided; 4 – partially agree; 5 – strongly agree). The scale is from 20 to 100 points. It is a reliable and accurate tool. In relation to the Polish version, Cronbach’s *α* coefficient is 0.73 for the general score; 0.55 for the “difficulty describing feelings” scale; 0.71 for the “difficulty identifying feelings” scale; and 0.51 for the “externally oriented style of thinking” scale.

### The Curious Experiences Survey (CES)

The Curious Experiences Survey (CES; Goldberg, 1999) was applied to investigate a tendency toward dissociation. The questionnaire allows to calculate the general level of tendency toward dissociation and the scales included therein: “amnesia,” “absorption,” and “depersonalization.” The survey comprises 31 test items. Each item has a five-degree Likert scale (1 – never happens to me; 2 – rarely happens to me; 3 – sometimes happens to me; 4 – often happens to me; 5 – always happens to me). The scale is from 31 to 155 points. Psychometric tests carried out for the original language version indicate that it is a reliable tool; Cronbach’s *α* coefficient for calculating reliability is 0.91 for the general scale; 0.75 for “amnesia”; 0.76 for “absorption”; and 0.88 for “depersonalization” scale.

### The Michigan Alcoholism Screening Test (MAST)

The Michigan Alcoholism Screening Test (MAST; Selzer, 1971) was applied to investigate the intensification of alcoholic behaviors. The questionnaire is a screening test; it comprises 24 questions to which a subject responds “yes” or “no.” Questions are valued 0–5 points. The score on a general scale is within a range from 0 to 53 points. Obtaining 5 or more points means the statement of alcoholism, according to DSM-IV-TR classification. MAST provides results of sufficient reliability for research purposes, but considerable caution is advised when applying the one (Shields et al., 2007). As indicated in the studies, results of MAST are less reliable in the case of women and non-clinical cases; however, applying this tool in a clinical group, as a quantitative indicator of alcohol addiction, is fully justified. Shields (2003) has validated methods for alcohol screening measures relative to current standards. Results suggest that the AUDIT, MAST, and SMAST are generally capable of producing scores of sufficient reliability for most basic research purposes, and individuals administering these measures can do so with confidence in such situations.

## Results

The statistical analysis, which allowed testing formulated hypotheses, was carried out in IBM SPSS Statistics program, release 24. The program was used to analyze basic descriptive statistics, due to which it was possible to examine the distribution of subsequent measured variables. The hypotheses were tested using a series of correlation analyses, analysis of variance (ANOVA), and mediation analyses using PROCESS macro by Hayes (2013). A typical threshold, i.e., α = 0.05 was an adopted significance level. The level of alexithymia in the examined group amounted to *M* = 51.29; *SD* = 14.00. The scores for individual scales were as follows: difficulty in describing emotions *M* = 14.58; *SD* = 4.72; difficulty in identifying emotions *M* = 18.50; *SD* = 6.61; externally oriented style of thinking *M* = 18.21; *SD* = 4.79. Dissociation general score amounted to *M* = 53.26; *SD* = 14.86; amnesia *M* = 11.83; *SD* = 3.70; absorption *M* = 25.95; *SD* = 7.78; depersonalization *M* = 15.49; *SD* = 5.36.

Before more advanced statistical analyses were started, the analyses were carried out with an aim to verify whether statistically significant differences exist between the group of alcohol addicts and the control group as far as alexithymia, dissociation, and attachment styles are concerned. For this purpose, for all analyzed variables, a single factor ANOVA was carried out in an inter-group schema. The analysis revealed significant differences in the general level of alexithymia; *F*(1.195) = 51.505; *p* < 0.001, *M* = 45.24; *SD* = 11.94, *M* = 58.01; *SD* = 13.03 and in subscales: difficulty in identifying feelings *F*(1.195) = 43.824; *p* < 0.001, *M* = 15.82; *SD* = 5.53, *M* = 21.46; *SD* = 6.42; difficulty in describing feelings *F*(1.195) = 36.671; *p* < 0.001, *M* = 12.84; *SD* = 4.46, *M* = 16.58; *SD* = 4.19 and in relation to externally oriented style of thinking *F*(1.195) = 27.589; *p* < 0.001; *M* = 16.58; *SD* = 4.34, *M* = 19.97; *SD* = 4.71. An analysis carried out in relation to dissociation revealed significant differences for the general level of dissociation; *F*(1.195) = 60.051; *p* < 0.001, *M* = 46.02; *SD* = 10.05, *M* = 60.22; *SD* = 15.23 and in relation to subscales: absorption *F*(1.195) = 33.163; *p* < 0.001, *M* = 22.90; *SD* = 7.01, *M* = 28.75; *SD* = 7.25, depersonalization *F*(1.195) = 54.601; *p* < 0.001, *M* = 12.98; *SD* = 2.69, *M* = 17.85; *SD* = 5.99, amnesia *F*(1.195) = 55.267; *p* < 0.001, *M* = 10.14; *SD* = 2.07, *M* = 13.63; *SD* = 4.19. In relation to attachment styles, significant differences were revealed for every investigated attachment style dimension: intimacy [*F*(1.195) = 22.450; *p* < 0.001] *M* = 3.71; *SD* = 0.65, *M* = 3.26; *SD* = 0.68; depend [*F*(1.195) = 28.283; *p* < 0.001] *M* = 3.47; *SD* = 0.81, *M* = 2.89; *SD* = 0.74; and anxiety [*F*(1.195) = 19.516; *p* < 0.001] *M* = 2.25; *SD* = 0.84, *M* = 2.80; *SD* = 0.91. Individuals, who are addicted to alcohol, achieve significantly lower scores in relation to the subscales of intimacy and depend, and significantly higher scores for the dimension of anxiety. In order to verify the hypothesis concerning the relation between attachment styles and a tendency toward an alcohol addiction, a categorical variable was created based on three attachment dimensions. Ten individuals were not qualified to any attachment style, and as a result, they were excluded from this part of analyses. The gathered data were analyzed with a single factor analysis of variance with a Welch’s adjustment, which revealed a significant effect of the style of functioning in a close relation on the level of alcohol addiction *F*(3;186) = 7.545; *p* < 0.001. For better understanding of the obtained result, a *post hoc* Dunnett’s test was performed additionally, based on the results of which a conclusion may be drawn that individuals from the secure attachment style (*M =* 12.11; *SD =* 17.49) evince a significantly lower level of alcohol addiction than individuals from the anxiety related attachment style (*M =* 30.68; *SD =* 19.45). Since no significant differences were stated in the addiction levels between other groups, an additional analysis of contrasts was performed where individuals from the secure attachment style were compared with other groups. The analysis revealed significant differences between the means *t*(186) = 3.897; *p* < 0.001. Individuals from insecure attachments (*M*_avoidancestyle_ = 17.73; *M*_absorbtionstyle_ = 18.83; *M*_anxietystyle_ = 30.68) reveal significantly higher level of addiction to alcohol than individuals from secure attachment styles. The subsequent stage of the analysis was to verify relations between the level of alexithymia and attachment styles, and the intensification of alcoholic behaviors. For this purpose, the *Pearson’s r* correlation analysis was performed. The results of analysis have been included in Table 1.

**Table 1:** The analysis of correlations between particular dimensions of attachment styles, alexithymia, and dissociation, and the intensification of alcoholic behaviors.

	Addiction to alcohol based on MAST

	***r*-Pearson**
Alexithymia – general score	0.473^∗∗^
Difficulty describing emotions	0.431^∗∗^
Difficulty identifying feelings	0.419^∗∗^
Externally oriented style of thinking	0.381^∗∗^
Intimacy dimension	–0.368^∗∗^
Depend dimension	–0.355^∗∗^
Anxiety dimension	0.293^∗∗^
Dissociation – general score	0.578^∗∗^
Amnesia	0.511^∗∗^
Absorption	0.492^∗∗^
Depersonalization	0.521^∗∗^


*^∗^p < 0.05; ^∗∗^*p* < 0.001.*

**FIGURE 1:**
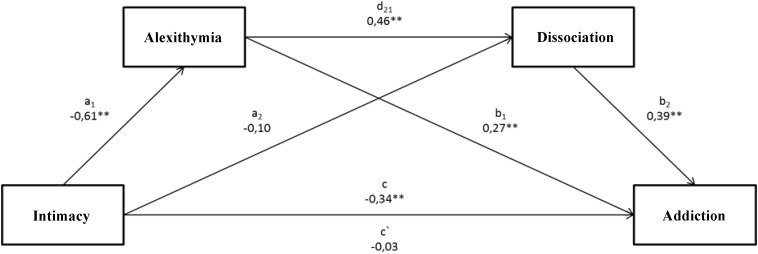
The mediational model for the relation between an intimacy dimension in the attachment style and the intensification of alcoholic behaviors, where alexithymia and dissociation constitute mediators. ^∗^*p* < 0.05; ^∗∗^*p* < 0.01. (c) A direct effect of the impact of intimacy on the addiction level. (a1, b1) An indirect effect of the impact of intimacy on the addiction level, including alexithymia. (a2, b2) An indirect effect of the impact of intimacy on the addiction level, including dissociation. (a1, d21, b2) An indirect effect of the impact of intimacy on the addiction level, including alexithymia and dissociation. (c′) A direct effect of the impact of intimacy on the addiction level, taking account of the impact of both mediators.

Based on obtained results, three mediation models were built, where the main independent variables were three attachment style dimensions, respectively: intimacy, depend, and anxiety. The mediators of relations included, respectively, alexithymia and dissociation, and the intensification of alcoholic behaviors constituted a dependent variable. In order to perform the mediation analysis with two mediators, a non-standard macro to SPSS Process by Hayes (2013) was applied. A bootstrapping method was used to analyze indirect effects, which allows to estimate the significance level with higher probability based on confidence intervals (Preacher and Hayes, 2008). The performed analysis of models related to the impact of intimacy on the intensification of alcoholic behaviors with regard to a mediatory role of alexithymia and dissociation (Figure 1) revealed that the tested model was statistically significant *F*(3.197) = 34.41; *p* < 0.001. The presented model explains 34% (*R*^2^ = 0.3438) of alcohol addiction scores’ variability. The analysis of indirect effects based on confidence intervals using 10,000 bootstraping and the correction on the significance level of 95% showed that the indirect effect of the impact of intimacy on the addiction to alcohol with the mediatory role of alexithymia (a1, b1) was significant (confidence interval 0.1606; 0.9130). As for the second indirect effect, where dissociation constitutes a mediator (a2, b2), the result turned out insignificant (confidence interval -0.0723; 0.3273). Turning finally to the last indirect effect of the impact of intimacy on the intensification of alcoholic behaviors with alexithymia and dissociation as mediators (a1, d21, b2), it was proven that this effect was significant (confidence interval 0.1579; 0.5946). The last important element is the change in the intensity of a dependency between intimacy and the intensification of alcoholic behaviors, which was statistically significant (c = -0.34; <-0.4668; -0.2034>), whereas after taking both mediators into account, the direct effect was insignificant (c′ = -0.03 < -0.1731; 0.1148>).

**FIGURE 2:**
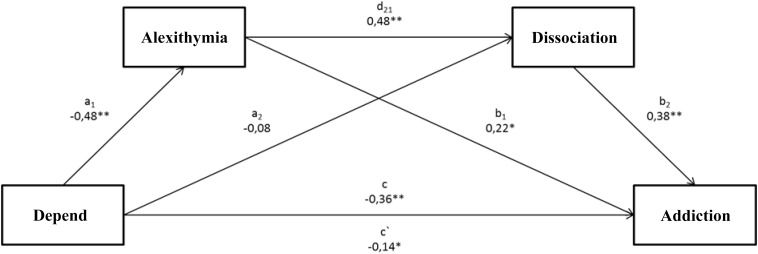
The mediational model for the relation between a depend dimension in the attachment style and the intensification of alcoholic behaviors, where alexithymia and dissociation constitute mediators. ^∗^*p* < 0.05;^∗∗^*p* < 0.01. (c) A direct effect of the impact of depend on the addiction level. (a1, b1) An indirect effect of the impact of depend on the addiction level, including alexithymia. (a2, b2) An indirect effect of the impact of depend on the addiction level, including dissociation. (a1, d21, b2) An indirect effect of the impact of depend on the addiction level, including alexithymia and dissociation. (c′) A direct effect of the impact of depend on the addiction level, taking account of the impact of both mediators.

The performed analysis of the model related to the impact of depend on intensification of alcoholic behaviors with regard to a mediatory role of alexithymia and dissociation (Figure 2) revealed that the tested model was statistically significant *F*(3.197) = 36.55; *p* < 0.001. The presented model explains 36% (*R*^2^ = 0.3576) of alcohol addiction scores’ variability. The analysis of indirect effects based on confidence intervals using 10,000 bootstraping and the correction on the significance level of 95% showed that the indirect effect of the impact of depend on the addiction to alcohol with the mediatory role of alexithymia (a1, b1) was significant (confidence interval 0.0764; 0.5730). As for the second indirect effect, where dissociation constitutes a mediator (a2, b2), the result turned out insignificant (confidence interval -0.0602; 0.2626). Turning finally to the last indirect effect of the impact of depend on the intensification of alcoholic behaviors with alexithymia and dissociation as mediators (a1, d21, b2), it was proven that this effect was significant (confidence interval 0.1254; 0.4239). The last important element is the change in the intensity of a dependency between depend and the intensification of alcoholic behaviors, which was statistically significant (c = -0.36; <-0.4890; -0.2280>), whereas after taking both mediators into account, the direct effect was still significant; however, the strength of a relation between depend and addiction to alcohol decreased (c′ = -0.14 < -0.2647; -0.0078>).

**FIGURE 3:**
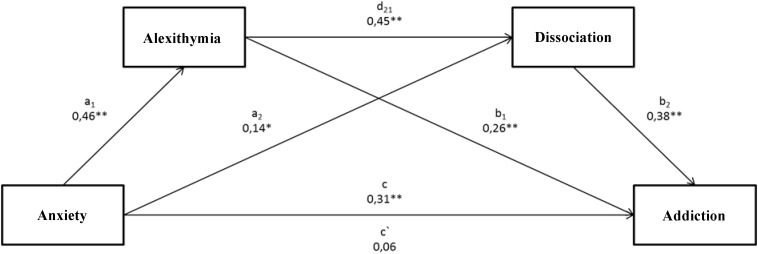
The mediational model for the relation between an anxiety dimension in the attachment style and the intensification of alcoholic behaviors, where alexithymia and dissociation constitute mediators. ^∗^*p* < 0.05; ^∗∗^*p* < 0.01. (c) A direct effect of the impact of anxiety on the addiction level. (a1, b1) An indirect effect of the impact of anxiety on the addiction level, including alexithymia. (a2, b2) An indirect effect of the impact of anxiety on the addiction level, including dissociation. (a1, d21, b2) An indirect effect of the impact of anxiety on the addiction level, including alexithymia and dissociation. (c′) A direct effect of the impact of anxiety on the addiction level, taking account of the impact of both mediators.

The performed analysis of the third model related to the impact of anxiety on the intensification of alcoholic behaviors with regard to a mediatory role of alexithymia and dissociation (Figure 3) revealed that the tested model was statistically significant *F*(3.197) = 34.71; *p* < 0.001. The presented model explains 35% (*R*^2^ = 0.3458) of alcohol addiction scores’ variability. The analysis of indirect effects based on confidence intervals using 10,000 bootstraping and the correction on the significance level of 95% showed that the indirect effect of the impact of anxiety on the addiction to alcohol with the mediatory role of alexithymia (a1, b1) was significant (confidence interval 0.1293; 0.8429). As for the second indirect effect, where dissociation constitutes a mediator (a2, b2), the result turned out insignificant (confidence interval -0.0065; 0.4159). Turning finally to the last indirect effect of the impact of anxiety on the intensification of alcoholic behaviors with alexithymia and dissociation as mediators (a1, d21, b2), it was proven that this effect was significant (confidence interval 0.1264; 0.5034). The last important element is the change in the intensity of a dependency between anxiety and the level of addiction to alcohol, which was statistically significant (c = 0.31; <0.1751; 0.4411>), whereas after taking both mediators into account, the direct effect was insignificant (c′ = 0.06 < -0.0726; 0.1861>).

## Discussion

The purpose of the study was to verify the assumptions related to the direct connection between dimensions of attachment styles in close relations in adulthood and the intensification of alcoholic behaviors. It was assumed that alexithymia and dissociation would be important mediators in this relation. As a result of performed mediation analyses separately for every dimension, it was proven that in relation to a dimension of intimacy, a direct effect of the impact on addiction decreases to the level which is statistically insignificant, if both mediators, alexithymia and dissociation, are taken into account simultaneously. The obtained result means that the higher level of alexithymia and an inclination to pathological dissociation, the greater fear of intimacy, and the higher intensity of negative emotions in a romantic relationship. It is worth noting that an indirect effect of alexithymia itself is significant in relation to intimacy on the intensification of alcoholic behaviors.

In the case of the second dimension of depend, the distribution of dependencies is slightly different. The lower degree of depend, the higher level of alexithymia and the stronger inclination to addiction. The higher level of alexithymia, the greater inclination to dissociation, and, at the same time, the impact of both variables increases the inclination to addiction. The mediation is of partial nature, the dimension of depend in a relationship is still significant as a factor related to the addiction, though the intensity of a direct effect is weaker. On the contrary, for a dimension of anxiety, it turns out that the higher anxiety, the higher level of alexithymia, and the higher intensification of alcoholic behaviors. Whereas the introduction of two mediators simultaneously causes the situation in which they take over the intensity of an impact on alcohol addiction, and the level of anxiety decreases to the level which is insignificant. Similarly as for the intimacy, the total mediation was shown since the intensity of the independent variable’s impact on a dependent variable decreased to an insignificant level.

Chronic dissociation and alexithymia contribute to difficulties in identifying feelings, chemically (alcohol-induced) transient dissociative states may be a paradoxical effort to identify and express feelings that are otherwise difficult to access. Alexithymia and dissociation may be regarded as processes which distort the perception of reality. The dissociation of mental processes relates, among others, to splitting of the stream of consciousness in threatening and possibly threatening situations, which may result in permanent personality development disorders (van der Hart et al., 2006). Alexithymia, on the other hand, by distorting proper recognition and understanding of emotions, including those expressed with face (Grynberg et al., 2012), effects the inappropriate use of emotions as important processes providing information on the mental condition and relationships with other people, and thereby contributes to the increase in the number of stress situations in romantic relationships. Alexithymia is also connected with the malfunction in the recognition of facial expressions, which results in deficits in creating semantic representations of terms related to emotions (Grynberg et al., 2012). Castellano et al. (2015) found in the meta-analysis that people with alcohol and substance use disorders had worse facial emotion recognition relative to controls. Donadon and Osório (2017) found that the alcohol-dependent individuals showed low accuracy in recognizing emotions as a whole and especially fear and disgust. In addition, the group needed greater emotional intensity to recognize joy, fear, disgust, and surprise. It also showed the increased reaction time for all emotions. The response time for surprise and the ability to recognize emotions such as fear and disgust was significantly associated with alcohol dependence.

The low level of intimacy and depend, and the high level of anxiety, supported by alexithymia and dissociation, result in interpersonal problems in romantic relations. It may be considered that being in a romantic relation constitutes, at the high level of dispositional alexithymia and dissociation, the source of continuous stress related to the generalized fear of intimacy and rejection experienced as the permanent mental tension. The focus on the experienced tension or stress hinders or even blocks verbalization and mentalization processes. Hence, some researchers treat alexithymia as an indication of affective mentalization disorders (Fossati et al., 2009). The mentalization is a process, which requires having cognitive representations of reality, and activating cognitive operations on those representations. A contradiction to this is undoubtedly “the poverty of dreams and fantasies, and a tendency to describe details related to a given episode which evoked emotions” (Sifneos, 1973). Functioning in an insecure relationship hinders the development of mentalization capabilities in child, and constitutes a twofold risk, i.e., provokes painful emotions and, at the same time, impedes the development of competences needed to regulate affect in adults. An insecure attachment hinders proper development, e.g., by evoking and maintaining long-term negative emotions, for which it is difficult to find relevant descriptions. When a caretaker, as an attachment figure, participates unpredictably in processes of arousal regulation in a child, they are not able to modulate it, and instead they lead to extreme levels of arousal stimulation, very high and/or very low, which are observed in persons who were abandoned or emotionally neglected in childhood (Main, 1996; Dozier et al., 1999; Liotti, 2004, 2009). As claimed by Allen et al. (2014), an attachment trauma favors the defensive withdrawal from the mental world, and, in the worst case, such withdrawal adopts the form of a phobic avoidance of mentalization. The mentalization deficits indeed block the process of overcoming life adversities, which is based on the reflection and self-control, in particular, in difficult, new, and stressful situations. Furthermore, it causes disturbances in building and implementing life plans, or satisfaction in a relationship. This aspect in individuals addicted to alcohol was pointed out in the studies by Thorberg and Lyvers (2006).

The present results thus reinforce the notion that alexithymia and dissociation are positively associated with the fear of intimacy, which in turn may account, at least in part, for the reported association between high levels of alexithymia and insecure attachment (Thorberg et al., 2011). Present findings are also consistent with the research by Montebarocci et al. (2004) and Besharat et al. (2014b) who showed positive relationships of TAS-20 alexithymia with the fear of intimacy, as well as insecure attachment and marital dissatisfaction (Besharat et al., 2014a). The consistent finding of a strong association between alexithymia, dissociation and the fear of intimacy suggests that targeting the latter to improve interpersonal functioning may be a viable approach in the treatment of clients suffering from depression, anxiety, or substance disorders, who also exhibit high levels of alexithymia.

## Conclusion

An important deficit, as far as alexithymia is concerned, is the absence of an ability to modulate affective processes by cognitive processes, which is reflected in disorders of experiencing, interpretation, and regulation of emotions. With high dispositional inclination toward dissociation, an additional disorder is observed of an accurate insight in individual’s own mental processes, which results in the accumulation of mental tension and the release of affect with stimulants, among others. Individuals with a higher level of alexithymia apply non-adaptive strategies for the regulation of affect in stressful situations, such as, for example, the suppression of emotions, to a greater extent than persons with a lower level of this feature. As a consequence, they feel stronger psychophysiological arousal and do not record changes in experiencing the negative affect. On the other hand, they apply less frequently adaptive strategies for regulating emotions, such as, e.g., cognitive reappraisal (Swart et al., 2009). Alexithymia and dissociation, since they constitute the strategies of invasive defense against high-stimuli situations, become a reason for the development of deficits which belong to the cognitive and affective dimension of emotional regulation. By blocking the processes of emotions’ identification and verbalization, they make the language not to serve for the purpose of changing self and others, since the communication is disturbed or distorted. It is difficult to reach an agreement in the absence of common meanings and accurate mutual understanding. Such processes may play an important role in the formation of various mental disorders, not only the addiction to alcohol, and may constitute a risk factor for their occurrence (Dubey and Pandey, 2013; Kret and Ploeger, 2015). As it was proven in the performed analyses, romantic relations based on high dispositional anxiety may be conductive to the development and maintenance of affective and cognitive deficits in the form of alexithymia and dissociation, and thereby emotional disorders in adulthood.

## Limitations

Limitations of our study include its correlational design and the use of only self-report measures. The scores obtained in our research are significant, but the variance under explanation is moderate. It means that in subsequent studies, it is necessary to take account of other factors, which might contribute to the fact that in regulating affect people resort to stimulants, including alcohol. Future research should be carried out to gather additional information on the regulation emotion strategy, self-differentiation, and emotional abilities in couples.

## Author Contributions

EZ-Ś made substantial contributions to the conception or design of the work or the acquisition. DŚ analyzed the data. EZ-Ś and DŚ interpreted the data for the work, drafted the work or revised it critically for important intellectual content, approved the final version to be published, and agreed to be accountable for all aspects of the work in ensuring that questions related to the accuracy or integrity of any part of the work are appropriately investigated and resolved.

## Conflict of Interest Statement

The authors declare that the research was conducted in the absence of any commercial or financial relationships that could be construed as a potential conflict of interest.
